# The right to die with dignity and conscientious objection

**Published:** 2015-06-30

**Authors:** Jose Henrique Rodrigues Torres

**Affiliations:** Professor of Criminal Law. Pontifícia Universidade Católica de Campinas . San Pablo. Brasil

 "*The project of life is linked to freedom, as a right of each person to choose their own destiny. (...) The project of life fully encompasses the ideal of the American Declaration (of the Rights and Duties of Man) of 1948, which proclaims the spiritual development as the supreme end and the highest expression of human existence*".^1^

Colombia's Constitutional Court, at guaranteeing the fundamental right to live and die with dignity, in the liberating expression of human rights, did not forget the mythical image of Charon ferrying the dead in his boat to Hades 2. In Colombia, the debate against death, which is stubborn and limitless, and contrary to the expression of the patients' will, cannot be accepted any longer as a duty or as a right of the doctors. Doctors must resign themselves to the conscious and independent decisions of their patients, understanding the dimension of existence and of human dignity and the limits of medicine and science. Physicians need to help lead their patients, with the necessary palliative care, in crossing the River Styx to the "world of the dead". Denying euthanasia, in terms of the decision of the Constitutional Court, constitutes a flagrant violation of a patient's "life project." Patients have, in the established circumstances, the right to legitimate anticipation of death 

 However, after this famous decision, in the midst of the front door of the right to live and die with dignity, an intransigent objector doorman, who ignoring its limits, went to resist the effective implementation of the right proclaimed by the Constitutional Court. Incidentally, in the Kafkaesque parable, at the front door of the Law, there was also a porter: a man asked to enter the law aimed to help him, but the porter did not allow him to enter, and the man, too weak to fight for his own rights, stood in the doorway, which was closed forever, and he could not get in 3.

 That is the reason why the Constitutional Court recently, reaffirming the fullness of the right to live and to die with dignity, ordered the national health system to take actions for the effective guarantee of this fundamental right 4. 

 Pursuant to that decision, the Ministry of Health and Social Protection issued Resolution 12.116 / 2015^5^ establishing criteria and procedures to ensure the effectiveness of the right to a dignified death, adopted specific rules to prevent the rule of consciousness objection: 

 • The members of the "Interdisciplinary Scientific Committees for the Right to Die with Dignity" cannot be objectors (Article 6, paragraph); 

 • and conscientious objection can only be exercised in writing and motivated by the doctors in charge of the implementation of the euthanasia procedure, allowing the IPS (the institutions that provide health services) that are entitled to the objection to provide the objector's substitution within 24 hours (Article 18). 

 It was necessary to deal with the consciousness objection and those rules were adopted with rigor. 

 It is true that in democratic states of law, the objection must be guaranteed as an expression of freedom of conscience, religious belief, or political or philosophical conviction. And it is also true that the UN General Assembly has already declared that freedom of belief is one of the cornerstones of a free and democratic society 6.

 However, this right is not absolute and cannot be exercised to prevent or derail the exercise of another fundamental right. No one can invoke conscientious objection to stop doing something or to escape an obligation. The objection is admitted, only when the objector can find an alternative to meet his/her legal obligations, guaranteeing the rights of third parties.

 Freedom of conscience, in the inner of each human being, is an impregnable and absolute right, which avoids any possibility of control. But not the freedom of its expression. Otherness is the limit for the objection. That is why the international guarantee system of Human Rights has set limits for objectors. The International Covenant on Civil and Political Rights, for example, guarantees freedom of expression (Article 19, 1), thought and religion (article 18, 1), as well as the right to not to be bothered because of personal opinions (Article 19, 1), but restricting the exercise of these rights, it states that they cannot prevail if there is the need to ensure the respect for the rights of others (Article 19, 3). Thus, in the spectrum of the guarantee system, when the right of the objector blocks the rights of third parties or violates an important social value, conscientious objection is unacceptable, especially when it is a professional obligation 7.

 Health professionals should be assured the right to practice their profession independently, so that it is not possible to force them to provide services that are contrary to their beliefs. However, if a patient's rights can be blocked by an objection, this will always be unacceptable.

 If, on the one hand, it is the State's duty to protect the fundamental right to die with dignity, and on the other hand, the respect for conscientious objection, it is the objector's duty not to harm others. The physician objector has a legal, ethical and professional responsibility to do good to patients and to respect their autonomy and "life project" 

 Under the aegis of hermeneutical criteria weighing, before the confrontation between the right to a dignified death and conscientious objection, a physician objector must, as well as other professionals in the health system, specifically substantiate the reasons for his/her objection. The objector must act immediately so that that right cannot be blocked and inform patients that they have their right to euthanasia, thereby guaranteeing access to the procedure by other professionals or institutions able to perform the act. The physician objector must lend full support until the procedure can be actually be performed and in exceptional situations, perform the procedure if there are no other health professionals available to do it. 

 Furthermore, the Colombian Constitutional Court, in another famous decision addressing the issue of conscientious objection, stated: "*With regard to individuals, it should be noted that conscientious objection refers to a religious conviction duly substantiated, and therefore it is not about jeopardizing the physician about whether he/she agrees or disagrees with abortion, nor may it involve ignorance of the fundamental rights of women; so, if conscientious objection is invoked by a doctor, he/she should immediately proceed to refer the woman who is under this hypothetical scenario to another doctor able to perform the abortion, notwithstanding that it can be subsequently found that the conscientious objection was admissible and relevant through mechanisms established by the medical profession*" 8 *.*

 Conscientious objection, as a right of the human person, as it has already been decided by the Constitutional Court in a similar case, cannot be exercised by the State or by the health institutions: "*conscientious objection is not a right of which the holders are legal entities or the state. It is only possible to recognize it to individuals, so that there can be no clinics, hospitals, health centers or whatever name they are called, to submit conscientious objection to the practice of abortion when the conditions in this judgment are met*" 8.

That is why the IPS are not worthy of conscientious objection and they have a duty to provide human and material resources so that the procedure requested can be conducted, fully and effectively, which demands the immediate replacement of the possible objectors, exactly as it is established in the resolution in question.

 As we can see, the Ministry of Health acted with rigor when it established these standards to face that doorman, conscientious objection, which cannot prevent patients from exercising their fundamental right to die with dignity.

 Norberto Bobbio is correct when he says that "today, the fundamental problem in relation to human rights is not to justify them, but to protect them; that is, it is a political problem, not a philosophical problem" 9.
